# Design and Fabrication of a Stacked Three-Phase Piezoelectric Composites Ring Array Underwater Ultrasound Transducer

**DOI:** 10.3390/ma14205971

**Published:** 2021-10-11

**Authors:** Lili Xia, Hongwei Wang, Qiguo Huang

**Affiliations:** School of Science, Beijing Information Science and Technology University, Beijing 100192, China; xll2004@126.com (L.X.); hjg303@163.com (Q.H.)

**Keywords:** underwater acoustic transducer, three-phase piezocomposites, ring array, broadband

## Abstract

A stacked three-phase piezoelectric composites ring array underwater ultrasound transducer was developed in this paper. The circular structure of three-phase piezoelectric composite with a large open angle was improved based on the 1-3 piezoelectric composites. The structure size of the transducer’s sensitive component was designed by using ANSYS simulation software, and the single-ring samples of three-phase piezoelectric composites with different thicknesses were fabricated. Based on the bandwidth broadening theory of multimode coupled vibration, the piezoelectric composite ring-shaped sensitive component was fabricated by the piezoelectric composite curved-surface-forming process. According to the design structure of the transducer, the stacked three-phase piezoelectric composites ring array underwater ultrasound transducer was processed. The experimental results show that the maximum transmission voltage response is 154 dB, the open angle of the horizontal beam reaches 360°, and the bandwidth of −3 dB is 86 kHz. The developed transducers achieved a high frequency, broadband, and large open angle to radiate sound waves.

## 1. Introduction

The underwater ultrasound transducer is the key device for energy conversion in the sonar system [1], and its performance affects sonar detection capability directly. Recently, water sonar equipment has widely demand small volume, light weight, high precision, and high resolution. Medium- and high-frequency transducers are used to complete the ship collision avoidance, underwater 3D imaging, underwater multi-beam scanning, seawater velocity measurement, and so on. Therefore, medium- and high-frequency transducers are usually used in the field of target detection and fine imaging [2] and the underwater acoustic transducer [3,4]. The high-frequency, broadband, and horizontal omnidirectional transducer is in urgent need of thunder-hunting sonar, anti-frogman sonar, seafloor-surveying sonar, near-field communication sonar, and forward-looking sonar of unmanned underwater vehicles [5,6].

The broadband and wide-beam properties are the two important performance indexes of underwater acoustic transducers. On the one hand, the high-frequency underwater acoustic transducer with broadband not only enables the transducer to transmit or receive more information but also significantly improves the resolution of the transducer array. It can also increase the detection distance of the system. S. Cochran et al. fabricated a transducer with a bandwidth of more than one octave by adding a matching layer to the 1-3 piezoelectric composites [7]. An electromechanical transducer employing axially symmetric vibrations of piezoelectric ceramic thin-walled tubes was presented based on the application of the energy method [8]. Refs. [9,10] studied the design of broadband longitudinal transducers and the wideband annular piezoelectric composite transducer configuration with a graded active layer profile, respectively. Zhang [11] gave a method to achieve the broadband-projecting performance of high-frequency transducers, and the bandwidth of 1-3 piezoelectric transducers can be expanded by the first thickness mode, the first lateral mode, and the third thickness mode. Huang et al. described the fabrication of 1-3 piezoelectric composites and prepared a high-frequency single-directional planar underwater ultrasound transducer [12]. The bandwidth of the transducer can also be extended by reducing the mechanical quality factor of the transducer piezoelectric element and combining it with the theory of multimode vibration coupling [13]. The mentioned transducer had expanded bandwidth. The researchers met the demand of improving the resolution of the transducer array. These results dealt less with the problem of enlarging the beam angle of the transducers.

On the other hand, the large beam opening angles can realize the transmitting or receiving of large open angle underwater acoustic signals. In terms of enlarging the transducer’s beam angle, the MSI company made six rows and four columns of an arc-shaped transducer array; its working range was 8–16 kHz, and the horizontal opening angle was 150° [14]. The design and performance of a high-frequency, wide-beam PVDF (polyvinylidene fluoride) ultrasonic projector were proposed for underwater communications in Ref. [15]. Deangelis and Schulze investigated the use of advanced Bode plot techniques to compare ultrasonic transducers with known “good” and known “bad” process performances, with the goal of a priori process assessment [16]. Hollow piezoelectric cylindrical shell transducers were made for underwater acoustic applications using suitable acoustical baffles, and the operational bandwidth was extended by using multiple resonant modes [17]. A vector hydrophone was developed for a towed array sonar system using a shear-type accelerometer. It has the highest feasible RVS (receiving voltage sensitivity) over the desired frequency range in comparison with other types of vector hydrophones such as the multimode hydrophone [18]. Teng et al. proposed an underwater, low-frequency, high-power transducer with a controllable transmitting beam by combining the directional beam transmission theory with the currently applied mosaic ceramic ring structure [19]. These results enlarged the beam angle of the transducers, but the bandwidth was relatively small.

**Figure 1:**
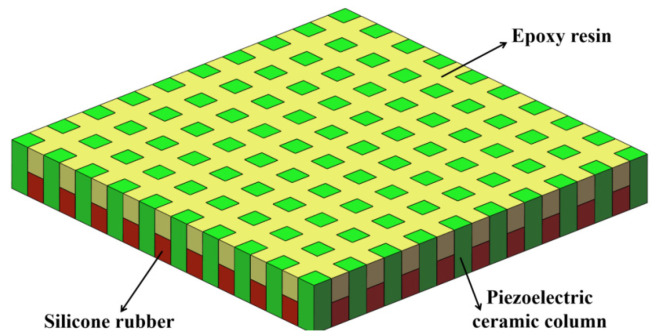
Planar three-phase piezoelectric composites structure.

In order to meet engineering requirements, scholars tried to fabricate the transducer with broadband and wide-beam properties. In order to expand the beam-width and bandwidth of a high-frequency transducer, a novel piezoelectric composite spherical cap vibrator was developed; the results show that the bandwidth of the transducer is 30 kHz and the −3 dB beam width is 40° [20]. At present, Wang et al. designed the sensitive element of an axially stacked piezoelectric composite ring array transducer by using finite element software [21]; the bandwidth of −3 dB can be expanded to 60 kHz by means of composites and multimode coupling. In the preparation of sensitive elements, the piezoelectric composite ring was made by cutting the piezoelectric ceramic ring radially and pouring epoxy resin into the slit. However, the process has the following disadvantages: (a) it is difficult to cut evenly along the radial direction of the ring, so the array elements are usually inconsistent; (b) the geometrical shape of the cut piezoelectric ceramic array is fan-shaped, which is not ideal; (c) the vibration modes are complex—the modal singleness and the flatness within the frequency band are both not good enough. The three-phase piezoelectric composite structure is proposed in this paper. The composite materials include piezoelectric ceramics, epoxy resin, and silicone rubber, which are called the three-phase piezoelectric composites. Figure 1 shows the planar three-phase piezoelectric composite structure. For the 1-3 type of composite materials, the three-phase piezoelectric composite overcomes the disadvantage of the two-phase (piezoelectric ceramics and epoxy resin) piezoelectric materials and improves the electromechanical coupling coefficient of the composite material. Compared with the pure piezoelectric ceramics, the quality factor of the material itself is reduced, the bandwidth is broadened, and the problem of the surface forming of the composite material is solved. This also meets the process requirements of the ring’s sensitive elements.

**Figure 2:**
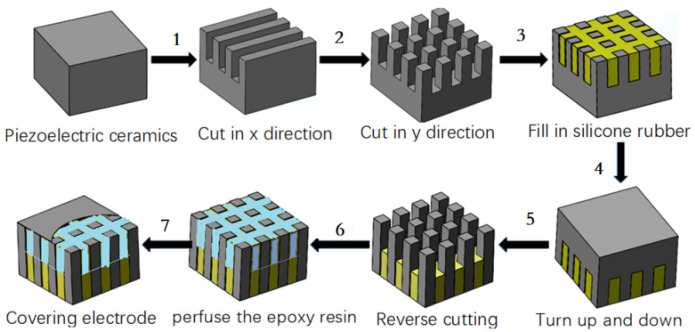
Fabrication flowchart of three-phase piezoelectric ceramic composites.

Different from the process in Ref. [21], the following five steps are carried out for the planar piezoelectric ceramic strip along the vertical length direction: (a) the ceramic strip is evenly cut on the front (not cut through); (b) silicone rubber is poured into the cutting seam; (c) the ceramic strip is cut reversely (cut through ceramic to silicone rubber); (d) silicone is added, and it is bent into a ring; (e) epoxy is perfused in the seam to cure it and then it is unmolded. The fabrication flowchart of the three-phase piezoelectric ceramic composite is shown in Figure 2. In Step 6 of Figure 2, it can be bent into a ring. The process of bending and forming a ring is shown in the double-ring-stacking process of Part 3.

It is called the curved-surface-forming process. This process can enlarge the beam opening angle. The improved three-phase composite structure can expand the bandwidth to a greater extent, and meanwhile, the spatial arrangement of piezoelectric ceramic small columns is consistent, so the sensitive element presents a single mode. The curved-surface-forming process is adopted in Ref. [22]; the bandwidth can be expanded by adding the matching layer to the single ring. In this paper, the underwater ultrasound transducer also adopted the curved-surface-forming process, but it is made up of a single circle longitudinally stacked with different thicknesses. A stacked three-phase piezoelectric composites ring array underwater ultrasound transducer is developed based on the theory of multimode coupling vibration and the curved-surface-forming process. A curved transducer with high frequency, wide band, and large beam opening angle is developed, which increases the detection accuracy and detection range of the sonar system.

## 2. Finite Element Analysis of the Three-Phase Piezoelectric Composite with a Double-ring Structure

**Figure 3:**
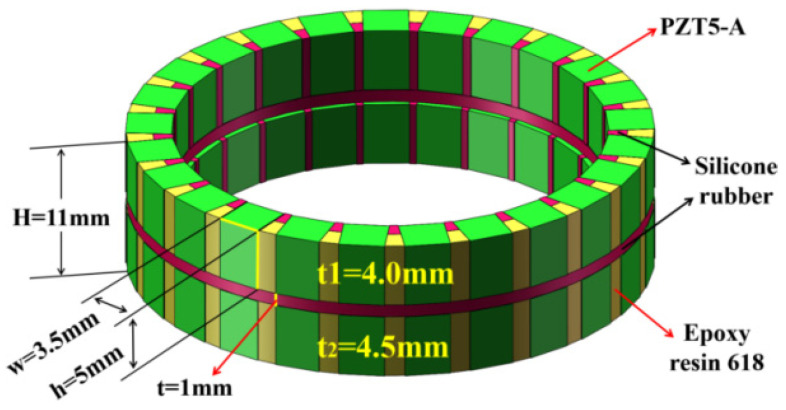
Structure of the double-ring stacked piezoelectric composite.

For the three-phase piezoelectric composite of a single ring, a periodic geometric unit is directly established, and then the geometric model of the composite ring can be obtained by copying the periodic unit through the COPY command [23,24,25]. On the basis of the advantage of a single ring, the double-ring piezoelectric composite is axially stacked by two single rings. Figure 3 shows the structure of the double-ring stacked piezoelectric composite. In this model, the height *h*_1_ and *h*_2_ of the two piezoelectric ceramic rings both equal 5 mm. The thickness of the top ring (*t*_1_) is 4 mm, the bottom ring (*t*_2_) isd 4.5 mm, and the interval between the two rings is 1 mm, so the total height is 11 mm. The width w of the piezoelectric ceramic cylinder in each ring both equal 3.5 mm, and each ring contains 25 identical piezoelectric ceramic cylinders. In this model, the piezoelectric ceramics accounts for 75% of the overall volume, and the polymer of silicone rubber and epoxy resin accounts for 25%. The piezoelectric composite with this volume ratio is beneficial to the emission performance of the underwater acoustic transducer. Due to the shape of the column itself, the transverse vibration of the column is more obvious, so the bandwidth can be expanded.

There are many methods to establish the model of ANSYS finite element simulation software [26,27]. In this paper, SolidWorks is used to draw a model diagram and import it into ANSYS finite element analysis software to complete the establishment of the finite element model.

**Figure 4:**
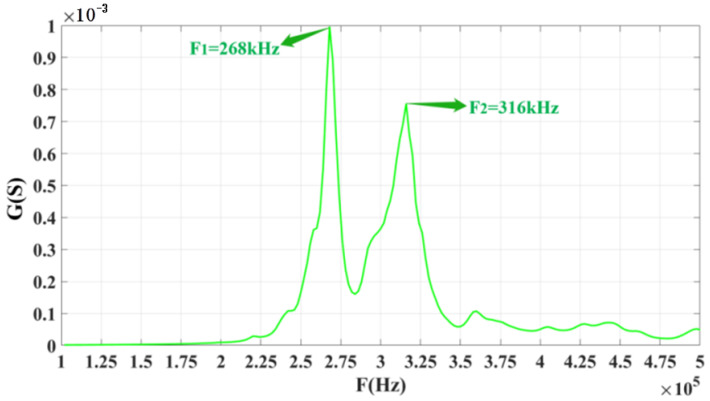
Conductance curves obtained from the simulation of the model.

The conductivity curve of the three-phase piezoelectric composite can be obtained after using ANSYS software to simulate the single array element of the double-ring stacked array. Figure 4 shows the conductance curve of the single array element. There are two peaks in the conductance curve of a single array element stacked from the figure. The frequency of the first peak is 268 kHz, and the second is 316 kHz. The center frequency between the two peaks is about 300 kHz.

**Figure 5:**
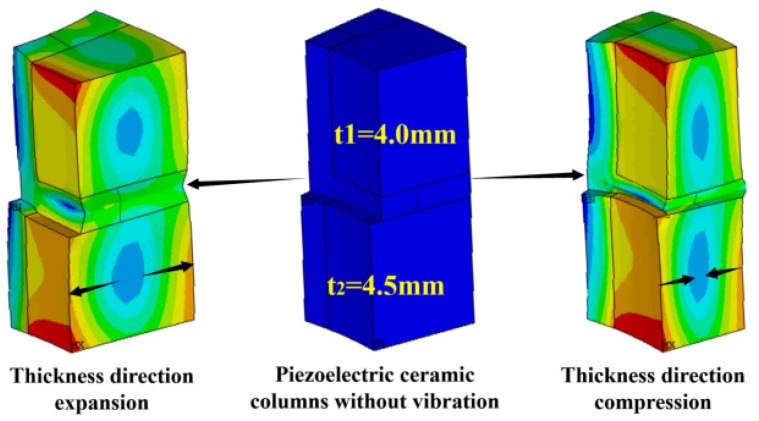
Different vibration modes of piezoelectric composite rings.

Figure 5 shows the vibration model of a single array element stacked with double rings. It can be seen from Figure 5 that the middle figure describes the periodic array element in the original state without voltage. The figure on the left shows the extended motion of the periodic array in the thickness vibration, while the right shows the compressed motion of the periodic array in the thickness direction. The results demonstrated that the surface displacement of the particle movement is not similar in the direction of compression and expansion, but there is a different state of concave and convex. This is why the piezoelectric cylinder is designed to be squat. It is also a design method for expanding the bandwidth of the transducer.

## 3. Fabrication and Testing of the Three-Phase Piezoelectric Composite’s Double Ring

**Figure 6:**
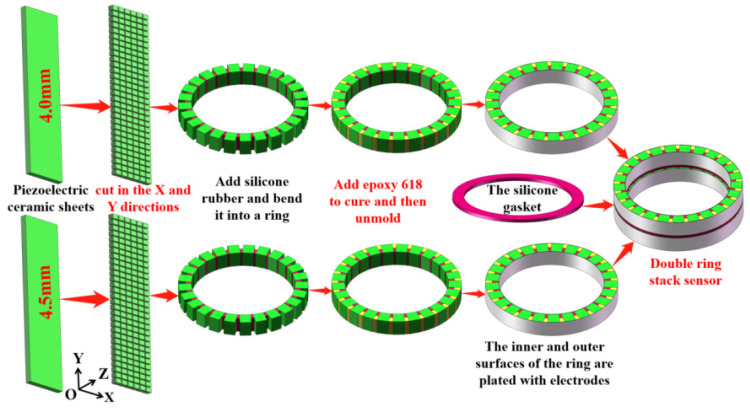
Fabrication flowchart of the double-ring stacked sensing element.

In order to fabricate the composite ring, the piezoelectric ceramic ring was firstly cut, then the epoxy resin was irrigated, cast, and cured, and finally, the piezoelectric composite ring electrode was fabricated. Figure 6 shows the fabrication flowchart of the double-ring stacked sensing element.

**Figure 7:**
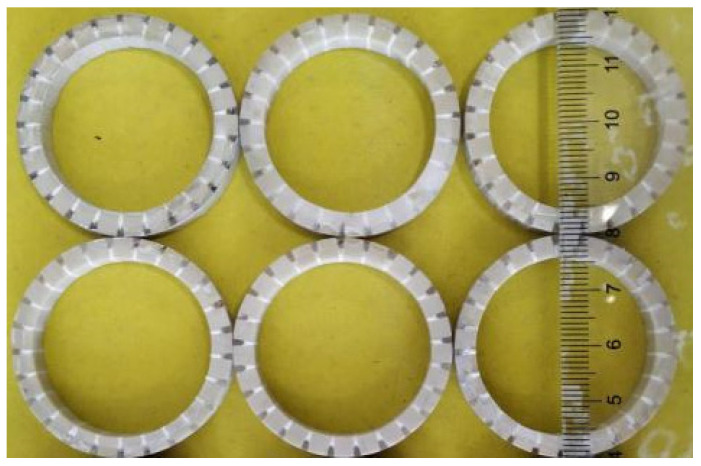
Three-phase piezoelectric composite single ring.

**Figure 8:**
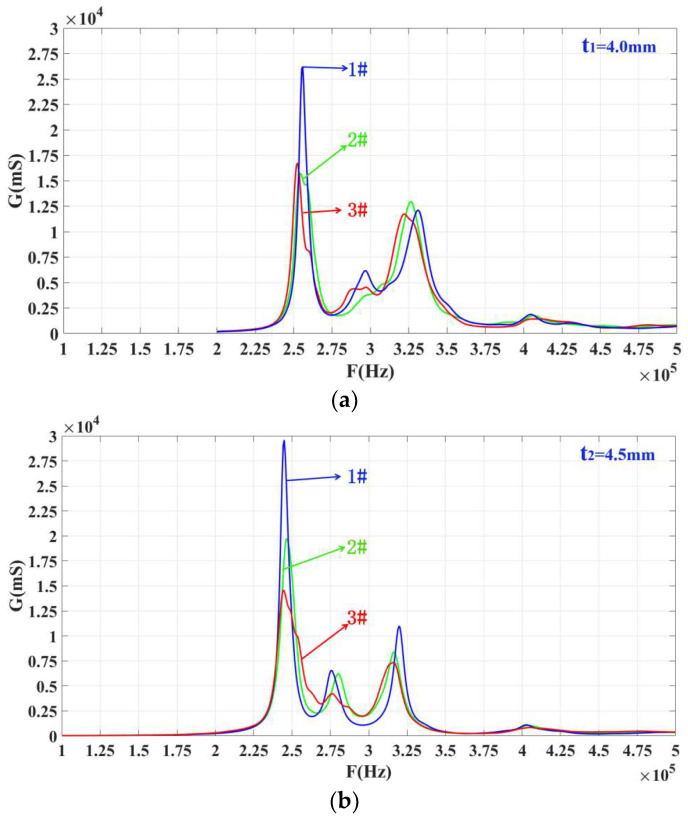
Conductance curves of sensitive components with two different thicknesses. (**a**) t_1_ = 4.0 mm, (**b**) t_2_ = 4.5 mm.

According to the strip-surface-forming process, samples of the three-phase piezoelectric composite single ring are shown in Figure 7. In Figure 7, two groups of three-phase piezoelectric composite single rings are fabricated, and each diameter is 20 mm. The first group consists of three rings with a thickness of 4 mm, and the second set of three rings is 4.5 mm. The conductivity curve of this single ring’s sensing element is tested. In order to compare the results of simulation analysis, the range of frequency scanning is set as 100–500 kHz, which is the same as the range set in simulation. The ring’s sensitive components are grouped and numbered, and the 4.0 mm and 4.5 mm thickness components are both divided into 1–3. The test results are shown in Figure 8.

From Figure 8, it can be concluded that each ring’s conductance curve has three different peaks. Each peak in the figure represents a modal of the system. The system has three modes, but the peak value in the middle (at 300 kHz for *t*_1_ = 4.0 mm samples and 275 kHz for 4.5 mm) is relatively small, and the modal can be ignored during analysis. The one on the left is larger than the right one. The peak frequency of the two experimental tests is closer to the simulation data of the ANSYS finite element analysis software. These two rings are selected for longitudinal stacking to fabricate a three-phase, piezoelectric composite, ring-stacking sensing element.

**Figure 9:**
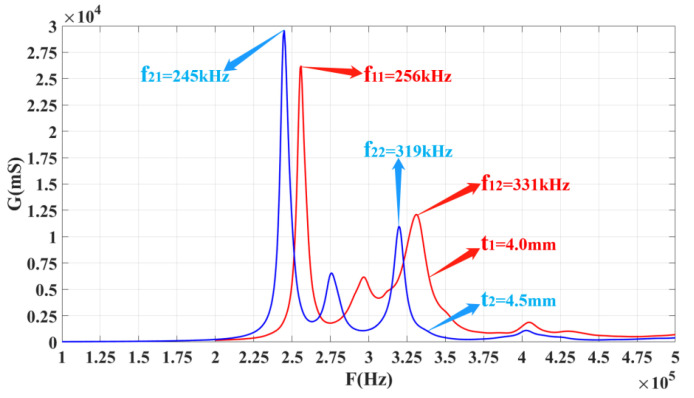
Conductance curves of every single ring of the stacked sensitive element.

**Figure 10:**
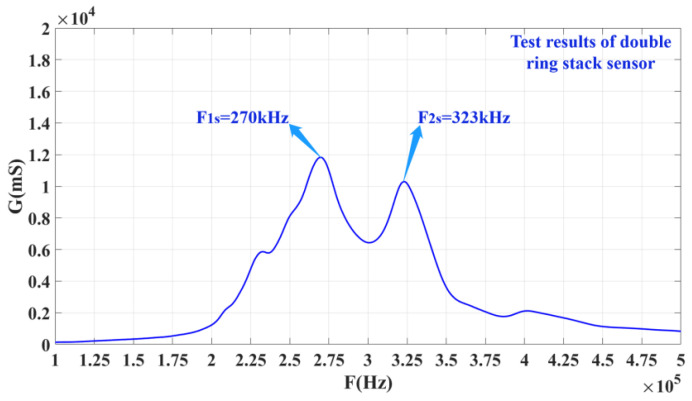
Conductance curves of the stacked sensitive element.

After the single ring is fabricated, it needs to be prepared as a sensitive element to conduct the conductivity curve and performance tests. Figure 9 shows the conductance curves of every single ring of the stacked sensing element. In this figure, *f*_21_ and *f*_11_ are close to each other, and these two peaks can realize multimode coupling after stacking, and the same for *f*_22_ and *f*_12_. Figure 10 shows a conductivity curve for the stacked sensing element. According to the test results in Figure 10, there are two peak frequencies on the conductivity curve of the sensor after stacking, and the frequency F_1s_ corresponding to the left peak is 270 kHz, while F_2s_ equals 323 kHz. The left and right peak values are coupled at around 300 kHz. For the conductance curves of the #1 single-ring sensing elements in Figure 8a,b, respectively, it is obvious that the left peak frequency F_1s_ is the result of the coupling between *f*_21_ and *f*_11_, and the right, *f*_2s_, is the result of mutual coupling between *f*_12_ and *f*_22_.

## 4. Structural Design and Performance Testing of the Transducer

**Figure 11:**
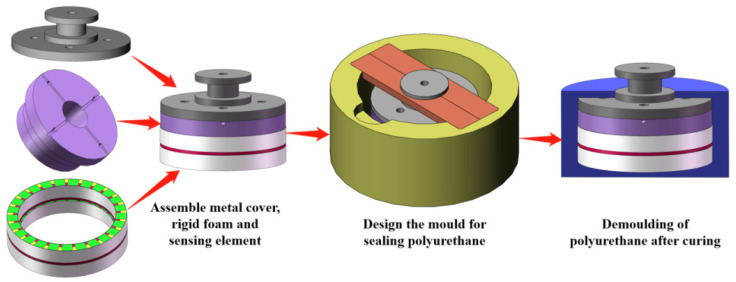
Assembly and the potting of the underwater acoustic transducer.

In the fabrication process, the key steps are the assembly of each component of the underwater acoustic transducer and the potting of the polyurethane waterproof sound permeable layer. Figure 11 shows the process flow chart of the assembly and potting of the underwater acoustic transducer.

**Figure 12:**
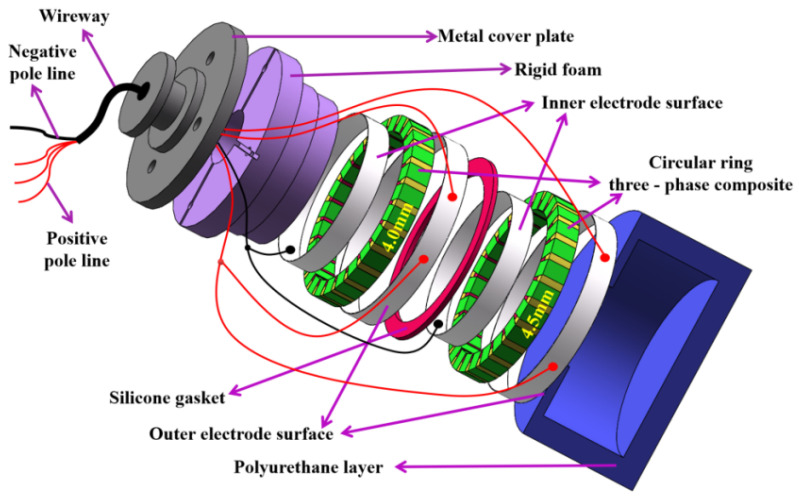
Structure of the array transducer.

**Figure 13:**
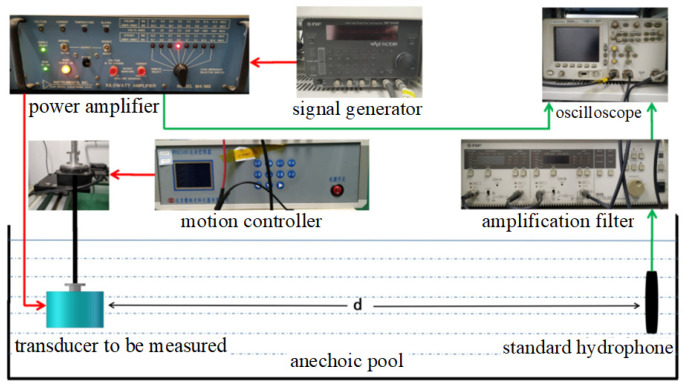
Underwater acoustic transducer test system.

The assembly and pasting method of each component of the high-frequency broadband directional cylindrical array transducer is shown in Figure 12. The assembly and pasting method of each component of the underwater ultrasound transducer is shown in Figure 13. The basic structure of the underwater acoustic transducer is composed of a polyurethane waterproof sound transmission layer, a stacked sensitive element, a positive and negative electrode lead, a hard foam, a metal cover plate, and a shielding wire. The positive and negative excitation ends of the AC signal are, respectively, connected to the positive and negative electric extremes of the underwater acoustic transducer. The sensitive element vibrates due to the action of the excitation signal, and the acoustic signal generated by the vibration will penetrate the sound-permeable layer of the polyurethane waterproof and propagate to the medium.

Underwater acoustic transducer performance testing needs to be carried out in an anechoic pool. The underwater acoustic transducer performance test system is shown in Figure 13, which consists of two parts, the transmitter part and the receiver part, respectively. The transmitter part contains a signal generator, a power amplifier, and a motion controller, while the receiver part contains an oscilloscope, an amplification filter, a power amplifier, and a standard hydrophone. The transmitting and receiving performance of the transducer can be measured using this device. Based on this testing system, the voltage response curve and opening angle of the underwater acoustic transducer are obtained, respectively.

**Figure 14:**
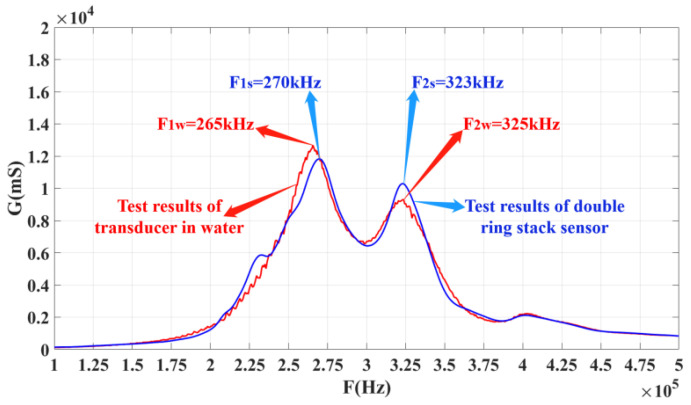
Conductance curve of the transducer in air and water.

Figure 14 shows the conductance curves of an underwater acoustic transducer in water and air. It shows that the two conductance curves of the hydroacoustic transducer are basically consistent. The conductivity curve of the underwater acoustic transducer shows two peaks when it is in the air. The left peak frequency, F_1s_, is 270 kHz, and the right peak frequency, F_2s_, is 323 kHz. When the transducer is placed in water, its conductivity curve becomes rough due to the fluidity of the water, and its conductivity is slightly deviated due to the load of the water. The peak frequency on the left, F_1w_, is 265 kHz, and the right, F_2w_, is 325 kHz. The conclusion is that the conductivity curve of the transducer in water tends to coincide with that of the stacked sensor before potting.

**Figure 15:**
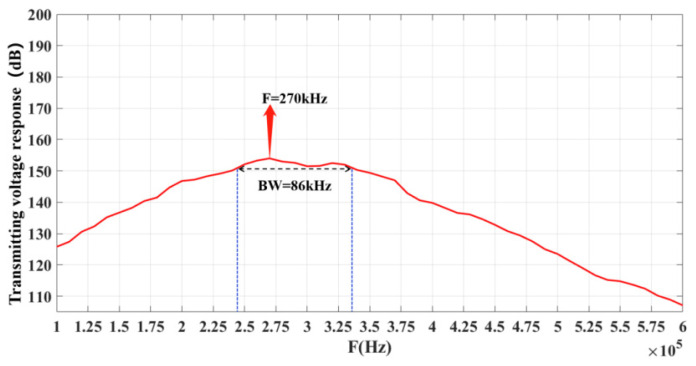
Response curves of transmitting voltage.

The voltage response curve of the underwater acoustic transducer is shown in Figure 15. The maximum transmission voltage response is 154 dB when the frequency of the transducer is 270 kHz. The performance test of the underwater acoustic transducer shows that the frequency range of −3 dB bandwidth of the transducer is 240–326 kHz, and the bandwidth is up to 86 kHz. Compared with the traditional composite transducer, its bandwidth has been significantly expanded. It also shows that the bandwidth of the transducer can be significantly expanded by the combination of composites and multimode coupled vibration.

**Figure 16:**
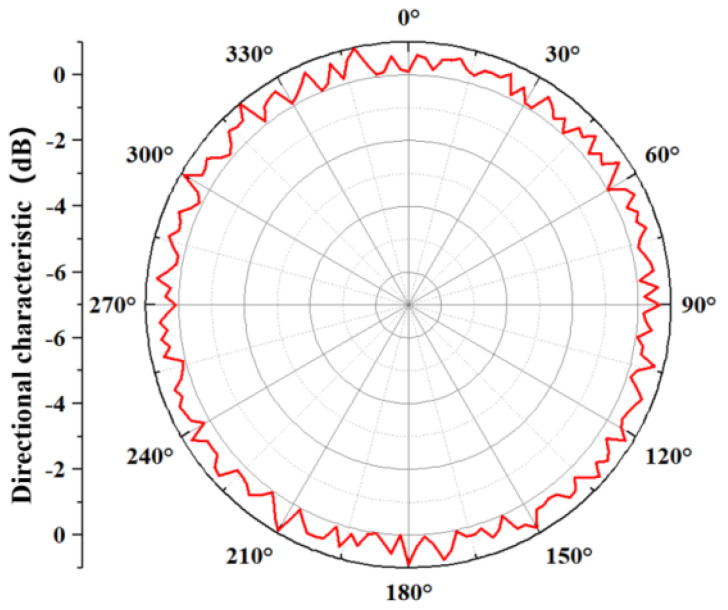
Directivity curve of transducer in the water.

The measurement result of the transducer’s directivity is shown in Figure 16. It is shown that the transducer has omnidirectional directivity in a one-dimensional direction, and the fluctuation is more stable within −3 dB. The directivity of the transducer realizes omnidirectional emission in the horizontal direction, which realizes the comprehensive coverage of radiated sound waves.

## 5. Conclusions

In this paper, the approximate model of the transducer is simulated using the ANSYS finite element analysis software. The fabrication process of the sensitive element and the potting technology of the transducer is introduced, and the transducer prototype is fabricated. An underwater acoustic transducer is fabricated with a working frequency band of 240–326 kHz, bandwidth up to 86 kHz, maximum transmission voltage response of 154 dB, and open angle of the horizontal beam of up to 360°. The goal of high frequency, wide band, and controllable directivity of the transducer has been achieved in this paper. In Table 1, the piezoelectric transducer studied in this paper is compared with other types of high-frequency transducers.

In Table 1, the following conclusions can be drawn: (a) compared with the current piezoelectric ceramic cylindrical transducer (transmission voltage response is about 140 dB and bandwidth is about 3–5 kHz), the transducer in this project can increase the transmitting voltage response by 14 dB and expands the bandwidth by 17; (b) compared with the piezoelectric ceramic PZT composite planar broadband transducer (about 150 dB transmitting voltage response and about 40 kHz bandwidth), the transducer in this project increases the transmitting voltage response by 4 dB and expands the bandwidth by about 46 kHz.

## Figures and Tables

**Table 1:** Comparison of various types of transducers.

Piezoelectric Transducer	Transmitting Voltage (dB)	Bandwidth (kHz)	Open Angle
Piezoelectric ceramic PZT cylindrical transducer	130~140	3–5	Horizontal omnidirectional
Piezoelectric ceramic PZT composite planar transducer (MSI)	150	40	<30°
Piezoelectric single-crystal PMNT vector hydrophone (Wilcoxon, USA)	-	-	<30°
Transducer in this paper	154	86	Horizontal omnidirectional

## Data Availability

The data presented in this study are available on request from the corresponding author.
